# Comparison of Clinical Profile between *P. vivax* and *P. falciparum* Malaria in Children: A Tertiary Care Centre Perspective from India

**DOI:** 10.1155/2014/132672

**Published:** 2014-04-09

**Authors:** Jagdish Prasad Goyal, Aarti M. Makwana

**Affiliations:** ^1^Department of Pediatrics, PDU Government Medical College, Rajkot, India; ^2^Department of Pediatrics, All India Institute of Medical Sciences, Virbhadra Road, Rishikesh 249201, India

## Abstract

*Background.* Malaria is a one of the leading causes of morbidity and mortality in tropical countries. *Plasmodium vivax* (*P. vivax*) is usually thought to be causing benign malaria with low incidence of complications as compared to *Plasmodium falciparum* (*P. falciparum*). *Methods.* This retrospective observational study included malaria patients who were admitted to K.T. Children Hospital and P.D.U. Government Medical College, Rajkot, a tertiary care teaching hospital, Gujarat, western India, during the period January 2012 to December 2012. Inclusion criteria were patients in whom either *P. falciparum* or *P. vivax* was positive on rapid malaria antigen test and peripheral blood smear. Patients showing mixed infections were excluded from study. *Results.* A total of 79 subjects (mean age 5.4 ± 3.6 years) were included in the study. It consisted of 47 *P. vivax* and 32 *P. falciparum* cases. The *P. vivax* cases consisted of 33 (70.2%) males and 11 (19.8%) females while *P. falciparum* cases consisted of 14 (43.8%) males and 18 (56.2%) females. One patient of each *P. vivax* and *P. falciparum* expired. There was no statistical significant difference found between complications such as anemia, thrombocytopenia, liver and renal dysfunction, ARDS, and cerebral malaria between *P. vivax* and *P. falciparum*. *Conclusion.* We conclude that *P. vivax* monoinfection tends to have as similar course and complications as compared to malaria due to *P. falciparum* monoinfection.

## 1. Introduction

According to the UNICEF, at every 30seconds, one child expires due to malaria [1]. It is one of the serious problems in our country due to inability to control disease in endemic areas, migration of the populations, and serious complication caused by the disease itself.* P. falciparum* malaria causes more severe disease, mortality and morbidity so intensive measures have been implemented mainly against it.* P. vivax* malaria has been neglected and mistakenly considered as “Benign” [2]. But there are few evidences in the past decade from studies in the countries of Asia that* P. vivax* is able to cause severe disease [3–5]. This may be due to its several important biological differences accounting for these observations, which are the development of the dormant stage in the liver (hypnozoites) causing relapse and greater transmission potential of* P. vivax* at low parasite densities.* P. vivax* is the most common geographically widespread species of* Plasmodium* causing malaria in human beings. The objective of this study was to compare the clinical profile and complications of* P. vivax* with* P. falciparum* malaria infection in children.

## 2. Subjects and Methods 

This retrospective observational study included malaria patients who were admitted to K.T. Children Hospital and P.D.U. Government Medical College, Rajkot, a tertiary care teaching hospital, Gujarat, Western India, during the period from January 2012 to December 2012.

Inpatient records from January 2012 to December 2012 were retrieved and scrutinized by using a Performa on the basis of the patient's demographic profile, clinical findings, investigations, treatment, and complications during this 12-month period. The institutional ethical committee approved the study. 

*Inclusion Criteria.* All slide positive and rapid diagnostic tests (RDT) that confirmed cases of malaria (*P. vivax* and* P. falciparum*) admitted and treated in K.T. Children Hospital with age group up to 12 years were included. 

*Exclusion Criteria.* These criteria included (1) patients presented with fever (smear negative for* P. vivax* and* P. falciparum* malaria) but treated empirically for malaria, (2) mixed infection of PF and PV malaria, and (3) patients presented with clinical features mimicking malaria like dengue fever, sepsis, meningitis were excluded from this study. We also excluded the newborn babies and those patients who died during resuscitation within the first hour in an emergency department before hospital admission formalities were complete.

The diagnosis and confirmation of species of* P. falciparum* and* P. vivax* malaria were established by thick and thin film of peripheral blood smear examination under oil immersion with giemsa stain and RDT [6]. The RDTs were based on detection of specific* Plasmodium* spp. lactate dehydrogenase (OptiMal test, Diamed AG, Cressier sur Morat, Switzerland) and histidine-rich protein 2 (Falcivax test; Zephyr Biomedical Systems, Goa, India). Severe complicated malaria was categorized as per World Health Organization guidelines. Severe complicated malaria in the form of cerebral malaria, severe anemia (Hb < 5 mg/dL), thrombocytopenia (platelet count < 1 lac/cumm), pancytopenia, jaundice (>3 mg/dL), acute renal failure (serum creatinine >3 mg/dL), gastrointestinal tract dysfunction, acute respiratory distress syndrome, and multiorgan dysfunction was included in this study. The level of consciousness was assessed using the modified Glasgow modified Glasgow Coma Scale in patients age <9 months and Glasgow Coma Scale in patients age >9 months. Routine laboratory investigations included a complete blood cell count, peripheral smear examination, blood indices, and platelet count and these were sent immediately after admission of all the patients. Urine examination, liver and renal function tests, coagulation profile, cerebrospinal fluid study, chest radiograph, and blood culture were done whenever it was indicated. The case definition of complicated and severe malaria was taken from the WHO guidelines for treatment of malaria 2010 [7]. Anemia in this study was defined when Hb of patient was ≤9 gm% while raised alanine aminotransaminase (ALT) was defined when ALT elevated >3x upper limit of normal.

### 2.1. Statistical Analysis

Statistical analyses were performed by social package for statistical science (SPSS) version 16. The data of the two groups were compared using the Fisher or chi-square test appropriate for each study parameter. Confidence interval and odds ratio of two groups were also reported.

## 3. Results

A total of 79 subjects (mean age 5.4 ± 3.6 years) were included in the study. It consisted of 47* P. vivax* and 32* P. falciparum* cases. Baseline characteristics of both groups were similar (Table 1). One patient of each of* P. vivax* and* P. falciparum* was expired. Fever was present in 100% of both* P. vivax* and* P. falciparum* cases. Anemia was present in 31.9% and 40.6% cases of* P. vivax* and* P. falciparum,* respectively, while thrombocytopenia was present in 36.1% and 36.7% cases. Raised liver enzyme and jaundice were present in 10.6% and 6.3% of* P. vivax* cases while the same were present in 6.2% and 9.3% cases of* P. falciparum*. ARDS was present in 4.2% cases of* P. vivax* and 3.1% cases of* P. falciparum*. Cerebral malaria was present in 4.2% and 6.2% of* P. vivax* and* P. falciparum* malaria cases, respectively.

Bivariate relationship between clinical features and complications of* P. vivax* and* P. falciparum* malaria showed no statistical significant difference (Table 2).

## 4. Discussion

In this retrospective observational study we report 79 patients with* P. vivax* and* P. falciparum* malaria. Clinical profile and complication of* P. vivax* were similar to those caused by* P. falciparum* malaria which included anemia, splenomegaly, thrombocytopenia, raised alanine aminotransferase (ALT), jaundice, renal failure, ARDS, and cerebral malaria. These findings of similar complication of* P. vivax* malaria were also reported by other authors [8, 9].

Thrombocytopenia is a well-known complication of* P. falciparum* malaria but also encountered in* P. vivax* malaria. This may be due to multiple factors which include increase in platelet destruction by platelet associated IgG antibody and its consumption [10, 11]. We observed thrombocytopenia in 36% of both* P. vivax* and* P. falciparum* malarial children. Other authors from India also reported significantly higher proportion of thrombocytopenia in* P. vivax*. [12, 13].

Raised liver enzyme and jaundice were present in 10.6% and 6.3% cases of* P. vivax *while the same were present in 6.2% and 9.3% cases of* P. falciparum*. Hepatic involvement has been well documented in* P. falciparum* malaria but also reported in* P. vivax* malaria. The possible explanation for hepatic involvement is direct injury to liver by parasite leading to malarial hepatitis [14–16].

Renal failure was encountered in 2.1% and 3.1% of* P. vivax* and* P. falciparum* cases in our study, respectively. Renal failure was observed commonly in* P. falciparum* but also has been reported in* P. vivax* malaria. Renal failure in malaria is caused by parasitized red blood cells leading to mechanical obstruction. Microcirculatory disorders, disseminated intravascular coagulation, fluid loss, and hypoxic or immune-mediated necrosis of renal tubules and glomeruli are the possible mechanisms that may be implicated in* vivax* infection [17–20].

Acute respiratory distress syndrome (ARDS) has been encountered in 3-4% cases of* P. vivax* and* P. falciparum* malaria. One patient of* P. vivax* died due to ARDS. It is known to occur in* P. falciparum* malaria due to sequestration but not reported in* P. vivax* malaria very often. The recent studies have shown that organ specific sequestration, cytokines, and nitric oxide production are mainly responsible for this complication of* P. vivax* malaria [21, 22].

Cerebral malaria has been observed in 4.2% of* P. vivax* and 6.2% of* P. falciparum* cases in our study. Cerebral malaria is a very severe complication and leading to one of the most common causes of mortality of malaria. One of our infected patients of* P. falciparum* malaria died due to cerebral malaria. Though exact pathogenesis of cerebral malaria in* P. vivax* remains unknown, few studies suggested that it might be due to sequestration and cytokine mediated cerebral injuries [23, 24].

The major limiting factor of our study was its retrospective nature and the small sample size. We believe that* P. vivax* malaria infection is often underestimated though complications and mortality are almost similar in* comparison* to* P. falciparum* malaria. Further large scale studies are required to know the exact pathogenesis of complications of* P. vivax* malaria. There is an urgent need of public health measures to estimate the burden of* P. vivax* malaria so that adequate planning and control measures can be taken against this emerging problem.

The present study concludes that* P. vivax* monoinfection tends to have as severe course and complications as compared to malaria due to* P. falciparum* monoinfection.

## Figures and Tables

**Table 1:** Baseline characteristics of patients.

Baseline characteristics	*P. vivax* (*n* = 47)	*P. falciparum* (*n* = 32)	All patients (*n* = 79)
Age (years, mean SD)	5.2 (3.5)	5.6 (3.7)	5.4 (3.6)
Gender (male/female)	33/11	14/18	47/29
Weight for age (*z* score < −2)	5 (10.6%)	4 (12.5%)	9 (11.3)
Duration of fever (days, mean SD)	5.1 (4.01)	5.4 (2.6)	5.2 (3.3)
Length of hospital stay (days, mean SD)	4.2 (2.47)	5.0 (3.1)	4.6 (2.8)
Hemoglobin (gm%, mean SD)	7.92 (2.26)	8.1 (2.6)	8.01 (2.4)
Blood sugar (mg%, mean SD)	81.3 (16.04)	89.2 (13.6)	85.2 (14.8)

**Table 2:** Comparison of various parameters between *P. vivax* (*n* = 47) and *P. falciparum* malaria (*n* = 32) at initial presentation.

Parameters	*P. vivax n* (%)	*P. falciparum n* (%)	Odds ratio	95% CI	*P* value
(1) Anemia	15 (31.9)	13 (40.6)	0.68	0.26–1.77	0.21
(2) Splenomegaly	21 (44.6)	16 (50)	0.80	0.32–2.01	0.32
(3) Thrombocytopenia	17 (36.1)	12 (36.7)	0.94	0.36–2.44	0.27
(4) Raised ALT	5 (10.6)	2 (6.2)	1.77	0.32–13.96	0.90
(5) Jaundice	3 (6.3)	3 (9.3)	0.66	0.10–4.09	0.64
(6) Renal failure	1 (2.1)	1 (3.1)	0.67	0.01–27.1	0.40
(7) ARDS	2 (4.2)	1 (3.1)	1.37	0.10–41.88	0.42
(8) Cerebral malaria	2 (4.2)	2 (6.2)	0.67	0.6–6.73	0.35
